# Evaluation of alterations in serum immunoglobulin concentrations in components of metabolic syndrome, obesity, diabetes, and dyslipidemia

**DOI:** 10.1186/s12872-019-01296-0

**Published:** 2019-12-30

**Authors:** Dee Lin, Mary Barna Bridgeman, Luigi Brunetti

**Affiliations:** 1 Health Outcomes Policy & Economics, Rutgers School of Public Health, Piscataway, NJ USA; 2 Ernest Mario School of Pharmacy, Rutgers, The State University of New Jersey, 160 Frelinghuysen Road, Piscataway, NJ 08854 USA; 3 RWJBarnabas Health, Robert Wood Johnson University Hospital, New Brunswick, NJ USA; 4 RWJBarnabas Health, Robert Wood Johnson Somerset, Somerville, NJ USA

**Keywords:** Immunoglobulins, Dyslipidemia, Metabolic syndrome, Cardiovascular disease, Obesity, Diabetes

## Abstract

**Purpose:**

Serum immunoglobulins (Igs) play a critical role in modulating the immune response by neutralizing pathogens, although little is known about the effect of Igs in development of atherosclerotic cardiovascular disease (ASCVD). Elevated serum Immunoglobulin A (IgA) concentrations have been identified in previous studies in populations with obesity and hypertriglyceridemia, whereas variable concentrations of Immunoglobulin M (IgM) have been observed in the setting of dyslipidemia.

**Methods:**

In this cross-sectional study, investigators examined the association of serum Ig concentrations with components of metabolic syndrome, including obesity, diabetes, and dyslipidemia. All consecutive adult patients aged 18 years or older discharged from two academic teaching hospitals with serum Immunoglobulin G (IgG) concentration measured during their admission were evaluated, with a total of 1809 individuals included and stratified into two groups: those with and those without dyslipidemia.

**Results:**

Mean IgG concentration in individuals with and without dyslipidemia was 997 ± 485 mg/dL and 1144 ± 677 mg/dL, respectively (*P* <  0.0001). After controlling for confounders in the generalized linear model (GLM), the least square mean IgG concentration in individuals with and without dyslipidemia was 1095 and 1239 mg/dL, respectively (*P* <  0.0001). The mean IgA and IgM concentrations were not significantly different in individuals with and without dyslipidemia both before and after adjusting covariates. After controlling for confounding variables, all three serum Ig concentrations were not significantly different in individuals with and without diabetes.

**Conclusion:**

Dyslipidemia was associated with a lower mean serum IgG concentration. No association with any serum Ig was indentified in individuals with diabetes. Exploration of the association between alterations in serum Igs and metabolic syndrome and the role of alterations of Ig concentrations in disease progression represents an important step in identification of appropriate targeted treatment options for reducing cardiovascular risk.

## Background

Cardiovascular disease (CVD) is the leading cause of death in the United States (US) and other Western countries [1]. Of every 10,000 all-cause deaths in the US, 23 are due to CVD each year. Although the CVD morality rate has declined in the past two decades due to advanced treatment options and improved disease awareness, the mortality and morbidity burden of CVD remains high. Based on the CVD mortality rate in 2013, more than 2000 Americans die each day due to CVD, and 35% of them have not yet reached the age of 75 [2, 3]. Understanding the pathophysiology, identification of risk factors, and potential treatment targets are essential to further reducing the consequences of disease; these areas have become a key focus for researchers and clinicians alike. Atherosclerotic cardiovascular disease (ASCVD) risk is associated with a variety of factors, with serum cholesterol representing an important modifiable risk [4]. Despite the development of cholesterol lowering therapies, a subset of patients with residual risk exist [5]. Identification of physiological factors associated with dyslipidemia may facilitate discovery of therapies with novel mechanisms of action and aid in the selection of optimal treatment as emerging pharmacologic treatment options become available.

Serum immunoglobulins (Igs) play a critical role in immune response by binding to presumed pathogens, including pathogenic bacteria and viruses, and leading to their neutralization [6]. Little is known about the role of Igs in metabolic physiology and whether these proteins provide protection against or contribute to ASCVD risk. A previous study designed to evaluate the association between serum concentrations of certain Igs and metabolic abnormalities demonstrated serum immunoglobulin A (IgA) concentrations to be significantly higher in subjects with abdominal obesity and hypertriglyceridemia. Besides IgA, the serum concentration of immunoglobulin M (IgM) was also found to be significantly lower in subjects with dyslipidemia [7]. Contrary to the aforementioned findings, another study reported a positive association with serum IgM concentration and elevated triglycerides and reduced high-density lipoprotein cholesterol (HDL-C) ratio in male patients [8]. Exploration of the association between Igs and metabolic syndrome and its particular role in the disease progression is an important step in further investigation. This study aims to describe the alteration of serum Ig concentrations in subjects with and without components of metabolic syndrome, including obesity, diabetes (DM), and dyslipidemia.

## Methods

### Data source

This cross-sectional study utilized data extracted from the electronic medical record and discharge database at two academic teaching hospitals. All consecutive adult patients (aged 18 years or older) discharged from January 1, 2009 to January 1, 2016 with a serum immunoglobulin G (IgG) concentration measured during the specified time frame were eligible for inclusion in the study. As a restrospective study using electronic helath record data, we had no control over the Ig assays utilized. Patients were excluded from the analytic dataset if they were pregnant, had a history of hematologic malignancy, or an autoimmune condition. All comorbidities were identified using International Classification of Diseases, Ninth Revision, Clinical Modification (ICD-9-CM).

### Statistical analysis

Statistical analyses were performed using SAS 9.4 (Cary, NC). Demographic and clinical characteristics, including Ig concentrations, were compared in patients with and without dyslipidemia, DM, or obesity, respectively. Deyo Charlson Comorbidity Index (DCI) score was calculated for each subject using ICD-9-CM codes applied to each patient. A continuous variable was created for DCI. Chi-square tests were used for categorical variables and analysis of variance tests were used for continuous variables to determine statistical association at a significance level of 0.05. To understand the association between each Ig concentration and metabolic syndrome, a generalized linear regression model (GLM) using the PROC GLM procedure in SAS was built to adjust for covariates. The Ig concentration was treated as a continuous dependent variable in the model. The primary covariates of interest were metabolic conditions (dyslipidemia, DM, and obesity). When testing for one metabolic condition, an interaction term was created for the other two metabolic conditions in the model. All demographic and clinical variables with a *P*-value less than 0.2 in the bivariate analysis were considered for inclusion in the final GLM. This conservative entry criterion allows more covariates to be considered. This technique facilitated selection of confounders to test further in the GLM and is commonly used to adjust for relevant covariates [9–12]. While a *p* <  0.2 in the bivariate was used to identify covariates for further testing, only variables with a *p* <  0.05 were included in the final GLM models. DCI was also tested as a confounder in the GLM and as a sensitivity analysis, individual components (comorbidities) of the DCI score were tested as confounders in the GLM model (see Additional file 1: Tables S1 and S2). The least square means was calculated for each Ig using the best fitting GLM model after adjusting for confounding variables as described above.

## Results

A total of 1809 individuals met study selection criteria. Demographic and clinical characteristics of this population are summarized in Table 1. The mean age of all individuals was 62 ± 19 years and the DCI score ranged from 0 to 17 with a median of 3. A total of 549 (30%) had a diagnosis of dyslipidemia. Compared to those without dyslipidemia, individuals with dyslipidemia were significantly older (70 vs. 59 years, *P* <  0.0001) and had higher DCI score (3.5 vs. 2.9, *P* <  0.0001). Race was significantly associated with risk of dyslipidemia (*P* = 0.0016). The prevalence of DM was 46% in the dyslipidemia cohort, compared to 28% in those without dyslipidemia (*P* <  0.0001). The prevalence of obesity for these two cohorts were 37 and 31%, respectively (*P* = 0.0073). The mean serum IgG concentration in individuals with and without dyslipidemia was 997 ± 485 mg/dL and 1144 ± 677 mg/dL, respectively (*P* <  0.0001). After controlling for age, sex, race, diagnosis of DM, obesity, interaction between DM and obesity, and DCI, the least square mean serum IgG concentration in individuals with and without dyslipidemia was 1095 and 1239 mg/dL, respectively (*P* <  0.0001; Table 2). The mean serum IgA and IgM concentrations were not significantly different in individuals with and without dyslipidemia both before and after adjusting covariates. In our sensitivity analysis where Ig concentrations were controlled for individual components of the DCI, heart failure, mild liver disease, and solid tumor had a significant association with Ig concentration. However, the results were in line to those observed after controlling for DCI and are provided in Additional file 1: Table S1.

**Table 1:** Comparison of demographic and clinical characteristics stratified by dyslipidemia

	Total	Dyslipidemia	No dyslipidemia	*P*-value
Age, years (SD)	62 (19)	70 (13)	59 (20)	< 0.0001
Female, n (%)	899 (50)	260 (47)	639 (51)	0.1894
BMI, kg/m^2^ (SD)	29 (13)	30 (14)	29 (13)	0.0727
Race				
White, n (%)	1181 (65)	378 (69)	803 (64)	0.0016
Black, n (%)	251 (14)	66 (12)	185 (15)	
Asian, n (%)	133 (7)	51 (9)	82 (7)	
Other, n (%)	244 (13)	54 (10)	190 (15)	
Comorbidities				
Diabetes, n (%)	603 (33)	254 (46)	349 (28)	< 0.0001
Obesity, n (%)	588 (33)	203 (37)	385 (31)	0.0073
CCI (SD)	3.1 (2.6)	3.5 (2.6)	2.9 (2.6)	< 0.0001
Immunoglobulin Concentrations				
IgA, mg/dL(SD)	260 (239)	251 (187)	264 (260)	0.2868
IgG, mg/dL(SD)	1099 (629)	997 (485)	1144 (677)	< 0.0001
IgM, mg/dL(SD)	112 (256)	106 (214)	115 (273)	0.5096

**Table 2:** Least square mean serum Ig concentrations for individuals with and without dyslipidemia

	Dyslipidemia^**^	No Dyslipidemia^**^	*P*-value
IgA LS Mean (mg/dL)	271	290	0.1390
IgG LS Mean (mg/dL)	1095	1239	< 0.0001
IgM LS Mean (mg/dL)	92	99	0.5097

**Least Square (LS)Mean concentrations after controlling for age, sex, race, diagnosis of DM, diagnosis of obesity, interaction of DM and obesity, and DCI

When looking into individuals with and without DM (Table 3), older age, male sex, and higher body mass index (BMI) were associated with a higher risk of DM (all *P* <  0.0005). The proportions of patients with DM were consistent across race groups. Patients with DM were at a higher risk for dyslipidemia and obesity, and had a higher DCI, compared to those without DM (all *P* <  0.0001). A lower IgM concentration was numerically observed in individuals with DM (*P* = 0.0736) before controlling for covariates. After controlling for age, sex, race, dyslipidemia, obesity, interaction of dyslipidemia and obesity, and DCI, all three Ig concentrations were not significantly different in individuals with and without DM (Table 4).

**Table 3:** Comparison of Demographic and Clinical Characteristics Stratified by Diabetes

	Total	Diabetes	No diabetes	*P*-value
Age, years (SD)	62 (19)	68 (14)	60 (20)	< 0.0001
Female, n (%)	899 (50)	264 (44)	635 (53)	0.0004
BMI, kg/m^2^ (SD)	29 (13)	31 (14)	28 (12)	< 0.0001
Race				
White, n (%)	1181 (65)	377 (63)	804 (67)	0.0884
Black, n (%)	251 (14)	82 (14)	169 (14)	
Asian, n (%)	133 (7)	56 (9)	77 (6)	
Other, n (%)	244 (13)	88 (15)	156 (13)	
Comorbidities				
Dyslipidemia, n (%)	549 (30)	254 (42)	295 (24)	< 0.0001
Obesity, n (%)	588 (33)	265 (44)	323 (27)	< 0.0001
CCI (SD)	3.1 (2.6)	3.8 (2.5)	2.8 (2.5)	< 0.0001
Immunoglobulin Concentrations				
IgA, mg/dL(SD)	260 (239)	267 (171)	256 (268)	0.3875
IgG, mg/dL(SD)	1099 (629)	1093 (572)	1102 (655)	0.7577
IgM, mg/dL(SD)	112 (256)	97 (134)	120 (301)	0.0736

**Table 4:** Least square mean serum Ig concentrations for individuals with and without diabetes

	Diabetes	No Diabetes	*P*-value
IgA LS Mean (mg/dL)**	280	279	0.9253
IgG LS Mean (mg/dL)**	1170	1162	0.8078
IgM LS Mean (mg/dL)**	85	105	0.1588

**Least Square (LS) Mean concentrations after controlling for age, sex, race, diagnosis of DM, diagnosis of obesity, interaction of DM and obesity, and DCI

Individuals of advanced age, female sex, and white race (all *P* <  0.005; Table 5) were more likely to be obese. Obese individuals were more likely to be those with dyslipidemia and DM (all *P* <  0.005), but had a similar DCI score compared to non-obese individuals. All three Ig concentrations were not significantly different in individuals with and without obesity (general linear model not performed as none of the Ig *P*-values were less than 0.2) and this was also confirmed when controlling for individual components of the DCI as described above (Additional file 1: Table S2).

**Table 5:** Comparison of demographic and clinical characteristics stratified by obesity

	Total	Obesity	No Obesity	*P*-value
Age, years (SD)	62 (19)	60 (17)	64 (19)	0.0002
Female, n (%)	899 (50)	321 (55)	578 (47)	0.0039
BMI, kg/m^2^ (SD)	29 (13)	39 (18)	24 (4)	< 0.0001
Race				
White, n (%)	1181 (65)	410 (70)	771 (63)	< 0.0001
Black, n (%)	251 (14)	81 (14)	170 (14)	
Asian, n (%)	133 (7)	15 (3)	118 (10)	
Other, n (%)	244 (13)	82 (14)	162 (13)	
Comorbidities				
Dyslipidemia, n (%)	549 (30)	203 (35)	346 (28)	0.0073
Diabetes, n (%)	603 (33)	265 (45)	338 (28)	< 0.0001
CCI (SD)	3.1 (2.6)	3.1 (2.5)	3.1 (2.6)	0.7438
Immunoglobulin Concentrations				
IgA, mg/dL(SD)	260 (239)	268 (275)	256 (220)	0.3667
IgG, mg/dL(SD)	1099 (629)	1093 (585)	1102 (649)	0.7552
IgM, mg/dL(SD)	112 (256)	102 (126)	117 (300)	0.2741

## Discussion

The key finding of this study is that patients with dyslipidemia had IgG concentrations significantly lower than those without dyslipidemia, before and after adjusting covariates that could potentially confound these results. Patients with and without dyslipidemia had comparable IgM and IgA concentrations and none of the studied Ig concentrations showed significant association with diagnoses of DM or obesity.

As previously mentioned, it is unknown whether antibody production and resultant serum Ig concentrations may have a protective or detrimental effect in development of ASCVD. Atherosclerosis is recognized as a chronic inflammatory condition, with evidence to suggest different Igs may be present in atherosclerotic plaques; nonetheless, there have been conflicting effects of different serum Igs observed in pre-clinical trials [13]. It is hypothesized that, as an inflammatory condition, the atherosclerotic lesion may result in monocyte and macrophage recruitment, along with neutrophil activation and the production of reactive oxygen species; B cell activation, in turn, may result in production of serum Igs, including IgG, which may serve to block the oxidative low density lipoprotein (LDL)-macrophage interaction or result in macrophage apoptosis and cause attenuation of atherogenesis or plaque healing [13–15]. IgG, the primary Ig subtype in human circulation, has been suggested to have proatherogenic effects, although the functional effect of alterations in serum concentrations of IgG on ASCVD remains to be understood [13, 16].

There are few data available evaluating Ig concentrations and their association with disease. Those that are available are limited in sample size and population. Sadanand and colleagues evaluated associations between antiphospholipid antibodies and dyslipidemia in patients with antiphospholipid antibody syndrome and found that abnormal lipid values were statistically associated with anticardiolipin IgM but not IgG concentration [17]. Turkoglu found that *Chlamydia pneumoniae* IgG and serum lipids concentrations were elevated together in patients with acute coronary artery diseases [18]. In 2016, Khamis et al. reported that patients with higher baseline serum IgG concentrations were associated with experiencing a lower risk of cardiovascular events, especially when attributed to coronary heart disease [odds ratio, 0.66; 95% confidence interval, 0.57, 0.76; *p* <  0.0001], and concluded that IgG concentrations could be used as a measurement to predict cardiovascular events or to improve existing cardiovascular risk-scoring models [16]. All three studies were conducted outside of the US. Compared to the existing evidence, our findings identify lower IgG concentrations in patients with dyslipidemia, a risk factor for cardiovascular events [19]. However, since this is a cross-sectional study, the causality between IgG concentrations and dyslipidemia remains unclear.

Nonetheless, it has been suggested that alterations in serum Ig concentrations may occur in the setting of chronic disease associated increasing the risk of development of ASCVD. For example, in another cross-sectional study, Guo and colleagues identified a reduction in serum IgG and IgM concentrations and increased IgA concentrations in individuals with DM, suggesting that there is an independent relationship between alterations in serum Ig concentrations and prevalence of DM among adults in China [20]. Further, in the EPIC-Norfolk study, it was identified that, among a cohort of initially healthy subjects, serum IgG and IgM autoantibody concentrations, along with apoB-immune complexes, were not necessarily independent risk factors for coronary artery disease (CAD) or CAD events, these humoral immune markers may serve to modify CAD risk attributed to elevated levels of oxidative biomarkers attributed to accelerating ASCVD progression [21].

This study provides evidence of an association between serum Igs concentration and metabolic syndrome in a US population. Nonetheless, the research methodology and study design have several limitations. First, data used in this study were collected from medical centers in which serum Igs were tested during patients’ hospitalizations. As a result, subjects were those who were acutely ill and, therefore, could not represent the general population. We acknowledge there is a possibility to selection bias, however, there was an attempt to exclude patients with comorbidities that would influence IgG levels, including malignancy, immunodeficiency, and other autoimmune conditions. If there was selection bias, this would likely be non-differential, biasing the results toward the null. As a cross-sectional study, our data are thus preliminary and require confirmation in larger prospective studies. Second, the reason why serum Ig concentrations were tested in these patients were unknown, and it was reasonable to assume that the test was ordered because the practitioner believed that the patient had a condition that could affect serum Igs. Although DCI was adjusted in the multivariate regression model, patients with and without dyslipidemia could have very different comorbidities [22]. Using a comorbidity summary score might not work as well as adjusting individual comorbidities; however, we performed a sensitivity analysis adjusting for individual compents which did not change the results [23]. In addition, the exclusion of patients with malignancy may limit the external validity. Subjects were from two regional medical centers in New Jersey, which further limited the generalizability to a diverse population in the US or abroad. Concurrent medication use was not adjusted in this analysis due to data limitation. Finally, we cannot exclude the possibility in variation of Ig measurements as the immunoassays used to measure Ig changed over the time period studied and may have differed between sites (a 7 year period). As this was not a prospective study, we had no control over the use of a standard assay methodology. However, we have no reason to believe that those with metabolic disease had Ig preferentially measured with one assay over another.

Despite these limitations, our study identifies an association between serum IgG concentration and dyslipidemia after controlling for confounding variables. Whether this alteration is a bystander or within the causal pathway remains to be determined; however, previous research has suggested a link between humoral immunity and cardiovascular disease [13]. Khamis and colleagues reported that low serum IgG concentration was correlated to worse cardiovascular outcomes after adjusting for risk factors including those found in the Framingham risk score [16]. Further, individuals in the highest IgG tertile had a 60% lower risk of coronary heart disease. There are preclinical data from an ApoE deficient murine model suggesting that high dose polyclonal IgG reduces atherosclerosis; therefore, it is plausible that individuals with low serum IgG may be more likely to display dyslipidemia [24]. Ultimately additional data from larger cohorts are needed to determine the relationship between serum Ig and cardiovascular risk.

## Conclusions

The correlation of the presence of humoral immune markers, such as alterations in serum Ig levels, with the development of components of metabolic syndrome may represent an important aspect of understanding the variables influencing ASCVD risk. Measuring serum Ig levels may represent a potential biomarker for earlier identification of individuals at risk for development of the metabolic syndrome, or in the evaluation of patients with ASCVD to help optimize treatment intensity for patients with obesity, dyslipidemia, DM, or other comorbidities. The findings of this research suggest that alterations in serum IgG levels may be found in individuals with components of metabolic syndrome, however, confirmation in larger prospectively designed observational studies are necessary to identify potential implications for patient care. Further, the various IgG subtypes additionally complicate the use of this substance of a biomarker for disease risk, but represent a provocative area of research potential for further elucidating underlying pathology.

## Supplementary information

**Additional file 1: Table S1.** Least square mean serum Ig concentrations for individuals with and without dyslipidemia. **Table S2.** Least square mean serum Ig concentrations for individuals with and without diabetes.

## Data Availability

The datasets generated and/or analysed during the current study are not publicly available due to patient privacy concerns but may be available from the corresponding author on reasonable request.

## Abbreviations

ASCVD: Atherosclerotic cardiovascular disease
CAD: Coronary artery disease
CVD: Cardiovascular disease
DCI: Deyo Charlson comorbidity index
DM: Diabetes mellitus
HDL-C: High-density lipoprotein cholesterol
ICD-9-CM: International classification of diseases, ninth revision, clinical modification
IgA: Immunoglobulin A
IgG: Immunoglobulin G
IgM: Immunoglobulin M
Igs: Immunoglobulins
LDL: Low density lipoprotein

## References

[1] Abubakar II, Tillmann T, Banerjee A (2015). Global, regional, and national age-sex specific all-cause and cause-specific mortality for 240 causes of death, 1990–2013: A systematic analysis for the global burden of disease study 2013. Lancet.

[2] Mozaffarian D, Benjamin EJ, Go AS (2016). Heart disease and stroke Statistics-2016 update: a report from the American Heart Association. Circulation.

[3] Atlanta GA (2012). Morbidity and Mortality Weekly Report. Prevalence of cholesterol screening and high blood cholesterol among adults.

[4] Goff DC, Lloyd-Jones DM, Bennett G, Coady S (2014). 2013 acc/aha guideline on the assessment of cardiovascular risk: a report of the american college of cardiology/american heart association task force on practice guidelines. Circulation.

[5] Karalis DG, Victor B, Ahedor L, Liu L (2012). Use of lipid-lowering medications and the likelihood of achieving optimal LDL-cholesterol goals in coronary artery disease patients. Cholesterol.

[6] Litman GW, Rast JP, Shamblott MJ (1993). Phylogenetic diversification of immunoglobulin genes and the antibody repertoire. Mol Biol Evol.

[7] Gonzalez-Quintela A, Alende R, Gude F (2008). Serum levels of immunoglobulins (IgG, IgA, IgM) in a general adult population and their relationship with alcohol consumption, smoking and common metabolic abnormalities. Clin Exp Immunol.

[8] Song K, Du H, Zhang Q (2014). Serum immunoglobulin M concentration is positively related to metabolic syndrome in an adult population: Tianjin chronic low-grade systemic inflammation and health (TCLSIH) cohort study. PLoS One.

[9] Concato J, Feinstein AR, Holford TR (1993). The risk of determining risk with multivariable models. Ann Intern Med.

[10] Irie M, Nakanishi R, Yasuda M, Fujino Y, Hamada K, Hyodo M (2016). Riks factors for short-term outcomes after thoracoscopic lobectomy for lung cancer. Eur Respir J.

[11] Milewski R, Milewska AJ, Wiesak T, Morgan A (2013). Comparison of artificial neural networks and logistic regression analysis in pregnancy prediction using the in vitro fertilization treatment. Grammar Rhetoric.

[12] Sherrod BA, Johnston JM, Rocque BG (2016). Risk factors for unplanned readmission within 30 days after pediatric neurosurgery: a nationwide analysis of 9799 procedures from the American College of Surgeons National Surgical Quality Improvement Program. J Neurosurg Pediatr.

[13] Tsiantoulas D, Diehl CJ, Wilztum JL, Binder CJ (2014). B cells and humoral immunity in atherosclerosis. Circ Res.

[14] Mayadas TN, Cullere X, Lowell CA (2014). The multifaceted functions of neutrophils. Annu Rev Pathol.

[15] Galkina E, Ley K (2009). Immune and inflammatory mechanisms of atherosclerosis. Annu Rev Immunol.

[16] Khamis RY, Hughes AD, Caga-Anan M (2016). High serum immunoglobulin G and M levels predict freedom from adverse cardiovascular events in hypertension: a nested case-control substudy of the Anglo-Scandinavian cardiac outcomes trial. EBioMedicine.

[17] Sadanand S, Paul BJ, Thachil EJ (2016). Dyslipidemia and its relationship with antiphospholipid antibodies in APS patients in North Kerala. Eur J Rheumatol.

[18] Turkoglu C, Sonmez E, Aydinli A (2004). Relationship between dyslipidemia, C-reactive protein and serological evidence of chlamydia pneumoniae in Turkish patients with coronary artery diseases. New Microbiol.

[19] Miller M (2009). Dyslipidemia and cardiovascular risk: the importance of early prevention. QJM.

[20] Guo X, Meng G, Liu F (2016). Serum levels of immunoglobulins in an adult population and their relationship with type 2 diabetes. Diabetes Res Clin Pract.

[21] Ravandi A, Boekholdt SM, Mallat Z (2011). Relationship of IgG and IgM autoantibodies and immune complexes to oxidized LDL with markers of oxidation and inflammation and cardiovascular events: results from the EPIC-Norfolk study. J Lipid Res.

[22] Johnson ML, Pietz K, Battleman DS (2004). Prevalence of comorbid hypertension and dyslipidemia and associated cardiovascular disease. Am J Manag Care.

[23] Austin SR, Wong YN, Uzzo RG (2015). Why summary comorbidity measures such as the Charlson comorbidity index and Elixhauser score work. Med Care.

[24] Nicoletti A, Kaveri S, Caligiuri G, Bariéty J, Hansson GK (1998). Immunoglobulin treatment reduces atherosclerosis in apo E knockout mice. J Clin Invest.

