# Supplementary material for: Evaluation of alterations in serum immunoglobulin concentrations in components of metabolic syndrome, obesity, diabetes, and dyslipidemia
Source: BMC Cardiovasc Disord. 2019 Dec 30;19:319. doi: 10.1186/s12872-019-01296-0 (PMC6936077; doi:10.1186/s12872-019-01296-0)
Supplement: Supplementary file 1 — Additional file 1: Table S1. Least square mean serum Ig concentrations for individuals with and without dyslipidemia. Table S2. Least square mean serum Ig concentrations for individuals with and without diabetes. [file 12872_2019_1296_MOESM1_ESM.docx]

Table S1. Least square mean serum Ig concentrations for individuals with and without dyslipidemia

|  | Dyslipidemia | No Dyslipidemia | P-value |
| --- | --- | --- | --- |
| IgA LS Mean (mg/dL) | 266 | 286 | 0.1031 |
| IgG LS Mean (mg/dL) | 1079 | 1230 | < 0.0001 |
| IgM LS Mean (mg/dL) | 93 | 99 | 0.6856 |

** LS Mean serum Ig concentrations after controlling for age, sex, race, diagnosis of DM, diagnosis of obesity, interaction of DM and obesity, individual components of DCI (heart failure, mild liver disease, and solid tumor).

Table S2. Least square mean serum Ig concentrations for individuals with and without diabetes

|  | Diabetes | No Diabetes | P-value |
| --- | --- | --- | --- |
| IgA LSMean (mg/dL) | 270 | 281 | 0.3693 |
| IgG LSMean (mg/dL) | 1134 | 1173 | 0.2213 |
| IgM LSMean (mg/dL) | 87 | 105 | 0.1799 |

** LS Mean serum Ig concentrations after controlling for age, sex, race, diagnosis of DM, diagnosis of obesity, interaction of DM and obesity, individual components of DCI (heart failure, mild liver disease, and solid tumor).
